# People have modest, not good, insight into their face recognition ability: a comparison between self-report questionnaires

**DOI:** 10.1007/s00426-020-01355-8

**Published:** 2020-05-20

**Authors:** Daisuke Matsuyoshi, Katsumi Watanabe

**Affiliations:** 1 Faculty of Science and Engineering, Waseda University, 3-4-1 Ohkubo, Shinjuku, Tokyo, 169-8555 Japan; 2 Araya Inc., ARK Mori Bldg, 1-12-32 Akasaka ARK Hills, Minato, Tokyo, 107-6090 Japan; 3 Quantum Life Science and Functional Brain Imaging Research, National Institute of Radiological Sciences, National Institutes for Quantum and Radiological Science and Technology, 4-9-1 Anagawa, Inage, Chiba, 263-8555 Japan; 4 Art and Design, University of New South Wales, Sydney, Australia

## Abstract

Whether people have insight into their face recognition ability has been intensely debated in recent studies using self-report measures. Although some studies showed people’s good insight, other studies found the opposite. The discrepancy might be caused by the difference in the questionnaire used and/or the bias induced using an extreme group such as suspected prosopagnosics. To resolve this issue, we examined the relationship between the two representative self-report face recognition questionnaires (Survey, *N* = 855) and then the extent to which the questionnaires differ in their relationship with face recognition performance (Experiment, *N* = 180) in normal populations, which do not include predetermined extreme groups. We found a very strong correlation (*r* = 0.82), a dominant principal component (explains > 90% of the variance), and comparable reliability between the questionnaires. Although these results suggest a strong common factor underlying them, the residual variance is not negligible (33%). Indeed, the follow-up experiment showed that both questionnaires have significant but moderate correlations with actual face recognition performance, and that the correlation was stronger for the Kennerknecht’s questionnaire (*r* =  − 0.38) than for the PI20 (*r* =  − 0.23). These findings not only suggest people’s modest insight into their face recognition ability, but also urge researchers and clinicians to carefully assess whether a questionnaire is suitable for estimating an individual’s face recognition ability.

## Introduction

Self-report measure is one of the important methodologies in many disciplines of psychology, such as educational, developmental, clinical, social, and personality psychology. Using questionnaires, researchers have quantified people’s insight into their skills, intelligence, cognitive ability, personality, or mood, and have created psychological models or theories. Despite the prevalence of self-report in psychological measurements, the correspondence between self-evaluations of ability and objective performance has been debated. Zell and Krizan (2014) synthesized meta-analyses across diverse disciplines and ability domains and reported that the mean correlation between ability self-evaluations and behavioral performance was moderate (*M* = 0.29). This finding suggests that people have only modest insight into their ability, perhaps reflecting not only the inaccuracy or imprecision of self-evaluations but also the biases (e.g., social desirability or self-esteem) inherent to self-report questionnaires (Choi & Pak, 2005).

Although the meta-synthesis indicated a moderate relationship between people’s insight into their ability and actual performance, recent studies have reported that people have good insight into their face recognition ability using the 20-item prosopagnosia index (PI20) (Livingston & Shah, 2017; Shah, Gaule, Sowden, Bird, & Cook, 2015; Shah, Sowden, Gaule, Catmur, & Bird, 2015). Shah et al. developed the PI20 to serve as a new self-report measure for estimating face recognition ability and developmental prosopagnosia (DP) risk, while criticizing a pre-existing questionnaire (Kennerknecht, Ho, & Wong, 2008), a 15-item questionnaire developed in a Hong Kong population (hereafter, HK questionnaire), on the grounds that it correlates poorly with objective face recognition performance (Palermo et al., 2017) (but see Johnen et al., 2014; Stollhoff, Jost, Elze, & Kennerknecht, 2011). However, although the PI20 was aimed to overcome the weakness of the HK questionnaire (i.e., it contains items irrelevant to face recognition and it has a ‘weak relationship’ to actual behavioral performance), its performance was not validated formally against the HK questionnaire. No direct comparison between the questionnaires was performed not only in terms of their relation to behavioral performance, but also their own relationship. Thus, whether the PI20 outperforms the HK questionnaire remains unclear.

Moreover, whether people have insight into their face recognition ability also remains to be investigated. Recent studies have reached different conclusions regarding the association between self-report and actual face recognition performance (Livingston & Shah, 2017; Palermo et al., 2017; Shah, Gaule, et al., 2015). Not only do they differ in the questionnaire used, but also in their participant demographics. Shah, Gaule, et al. (2015) reported that people have good insight into their face recognition ability (*r* =  − 0.68); they used PI20 and recruited individuals ‘identified themselves as suspected prosopagnosics’ in addition to a normal population. On the other hand, Palermo et al. (2017) reported that people have moderate insight into their face recognition ability (*r* =  − 0.14); they used the HK questionnaire and recruited a normal population, without ‘suspected prosopagnosics’. (The distinction between ‘good’ and ‘moderate’ insight has been arbitrary and seems to be based solely on researchers’ intuition or convention without clarifying the criteria, but here we regard a significant correlation coefficient of *r* = 0.5 or larger as ‘good’ insight and a significant correlation coefficient less than *r* = 0.5 as ‘moderate’ or ‘modest’ insight.) These inconsistent results are likely to result from the two methodological differences. First, although the PI20 and the HK questionnaire are so similar and simply asking how good (or bad) people are at recognizing faces, their subtle differences in texts might lead to a difference in correlation between self-report and behavioral performance. Second, because Shah and colleagues used an extreme group approach (i.e., recruited ‘suspected prosopagnosics’), which almost always leads to upwardly biased estimates of standardized effect size (Preacher, Rucker, MacCallum, & Nicewander, 2005), they might observe an inflated correlation between self-report and behavioral performance. Thus, it is crucial to use the two questionnaires in the same population and assess the relationship between the questionnaires and their relation to behavioral face recognition performance. We examined this issue by administering the two questionnaires to a large population and performing a set of analyses including correlation analysis, hierarchical clustering, a brute-force calculation/comparison of reliability coefficients, and a behavioral validation using Taiwanese Face Memory Test (TFMT) (Cheng, Shyi, & Cheng, 2016), an East Asian version of Cambridge Face Memory Test (CFMT) (Duchaine & Nakayama, 2006). If the PI20 is a better self-report instrument in estimating face recognition ability than the pre-existing HK questionnaire, the PI20 is expected to have distinct or more desirable features (i.e., low or moderate correlation between the questionnaires, PI20-specific cluster, or higher reliability) and a greater prediction accuracy of behavioral face recognition performance compared to the HK questionnaire.

## Survey

### Materials and methods

#### Participants

All participants were recruited from job and volunteer web sites for students in Tokyo area. The recruitment advertisement did not ask whether they have difficulty recognizing faces. Neither inclusion nor exclusion criteria are related to self-reported face recognition ability. Eight hundred and fifty-five young Japanese adults [427 female, 428 male; mean age: 20.9 ± 2.2 (± 1 SD) years; range 18–36 years] participated in the survey along with another psychological experiments (not including the follow-up Experiment) and received monetary compensation for their 3-h participation [3000 yen (approx. US $30)]. All had normal or corrected-to-normal vision and none reported a history of neurological or developmental disorders.

### Procedure

We asked participants to complete the questionnaires using an 8-in. touchscreen tablet PC in the laboratory. They were required to indicate the extent to which 36 items (15 from the pre-existing Hong Kong (HK) prosopagnosia questionnaire (Kennerknecht et al., 2008), and 20 from the PI20 (Shah, Gaule, et al., 2015), and an additional item pertaining to self-confidence in face recognition ability: “I am confident that I can recognize faces well compared to others”) described their face recognition experiences. Responses were provided using a five-point Likert scale ranging from 1 (strongly disagree) to 5 (strongly agree). The participants were instructed to complete the questionnaires at their own pace. The questionnaire took about 5 min to complete.

### Data analysis

Because the HK questionnaire developed by Kennerknecht et al. (2008) contains four dummy questions (HK#10, #11, #12, and #13) that are irrelevant with respect to face identity recognition, we excluded these items and calculated the total scores ranging 11–55, using the remaining 11 items (hereafter, ‘HK11’) (score range 11–55). The four dummy items consisted of three items related to face *processing* [ability to judge facial gender (HK#10), facial attractiveness (HK#12), and facial emotion (HK#13)], but not pertaining to their own face *identity recognition* abilities, and one item not at all related to face recognition [spatial navigation deficits (HK#11)].

PI20 scores were calculated using all 20 items and ranged from 20 to 100. As females have been shown to exhibit superior performance in behavioral face recognition studies (Shapiro & Penrod, 1986), we examined sex differences between the questionnaire scores. In addition, we used polychoric correlation coefficients to infer latent Pearson correlations between individual items from the ordinal data. The polychoric correlation matrix was estimated using two-step approximation (Olsson, 1979).

Cronbach’s *α* and Revelle & Zinbarg’s omega total coefficients were calculated to assess the scale reliability of both HK11 and PI20. Omega total coefficients were estimated using a maximum likelihood procedure (Revelle & Zinbarg, 2009). Confidence intervals (CI) for the coefficients were estimated using a bootstrap procedure (10,000 replications) with a bias-corrected and accelerated approach (DiCiccio & Efron, 1996; Kelley & Pornprasertmanit, 2016).

As it was possible that higher reliability coefficients merely reflected the higher number of items in the PI20, relative to that in the HK11 (Cortina, 1993), we performed a brute-force calculation of reliability coefficients for all 167,960 (_20_C_11_) possible combinations of PI20 items taken 11 items at a time (i.e., subsets of the PI20 generated by choosing 11 of the 20 items), which allowed us to compare reliability coefficients between the questionnaires with a virtual match of the numbers of items.

### Results

#### Total scores and score distribution

**Fig. 1:**
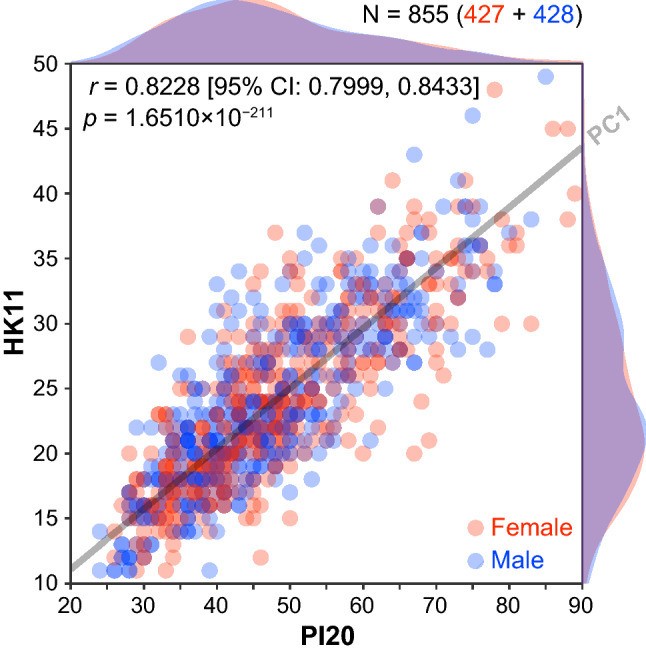
Correlation between total scores for the two prosopagnosia questionnaires (Survey). Scatter plot with color-coded transparent density curves of total scores for the 20-item prosopagnosia index (*x*-axis) and 11 items from Hong Kong prosopagnosia questionnaire (*y*-axis). Dots represent individual data, and color represents sex (red, female; blue, male). The gray transparent line represents a linear orthogonal regression line (first principal component, PC1 axis), which accounts for more than 90% of the total variance in scores in PCA with a singular value decomposition

Table 1 shows descriptive statistics for the total HK11 and PI20 scores. Independent two-sample t tests showed no significant differences in HK11 [*t*_853_ = 0.0511, *p* = 0.9592, Cohen's *d* = 0.0035 (95% CI − 0.1306, 0.1376)] or PI20 [*t*_810_ = 0.9578, *p* = 0.3384, Cohen's *d* = 0.0655 (95% CI − 0.0686, 0.1996)] scores between males and females. In addition, a Bayesian analysis using a JZS prior (*r* scaling = 1) (Rouder, Speckman, Sun, Morey, & Iverson, 2009) showed strong evidence for the null hypothesis (i.e., no sex difference) for both HK11 (Bayes factor BF_10_ = 0.0544) and PI20 (BF_10_ = 0.0856) scores. In addition, two-sample Kolmogorov–Smirnov tests showed no significant sex differences between the distributions (Fig. 1) of HK11 (*D* = 0.0265, *p* = 0.9982) and PI20 (*D* = 0.0460, *p* = 0.7554) scores. These results indicate that females and males showed almost identical mean HK11 and PI20 scores and score distributions, suggesting that sex was not a significant factor.

**Table 1:** Descriptive statistics of total scores for the questionnaires (*N* = 855, survey)

Questionnaire	Sex	Mean	SD	Min	Median	Max
HK11	Female	24.05	6.72	11	23	48
	Male	24.03	6.72	11	23	49
	Total	24.04	6.71	11	23	49
PI20	Female	48.33	13.00	26	46	89
	Male	47.49	12.70	24	46	85
	Total	47.91	12.85	24	46	89

A higher score indicates lower self-reported face recognition skills. Note that females and males showed similar total scores in terms of not only summary statistics, but also distribution, as shown in Fig. 1

#### Correlations between total scores

The results showed a very strong significant correlation between the total scores for the two questionnaires [Fig. 1, *r* = 0.8228 (95% CI 0.7999, 0.8433), *p* = 1.6510 × 10^−211^], suggesting a significant overlap of face recognition abilities assessed via each measure. It should be noted that Fisher’s *z* test with Zou’s CI (Zou, 2007) showed no significant sex difference in the correlation between total scores [*r*_diff_ =  − 0.0065 (95% CI − 0.0502, 0.0371), *z* = 0.2917, *p* = 0.7705; *r*_females_ = 0.8200 (95% CI 0.7863, 0.8489), *p* = 4.7087 × 10^−105^; *r*_males_ = 0.8265 (95% CI 0.7939, 0.8543), *p* = 2.3806 × 10^−108^]. Principal component analysis (PCA) with singular value decomposition of the correlation matrix between total scores showed that the first principal component (PC1) accounted for 91.1% (using standardized scores) and 94.2% (using raw scores) of the total variance in scores.

#### Correlations between individual item scores

**Fig. 2:**
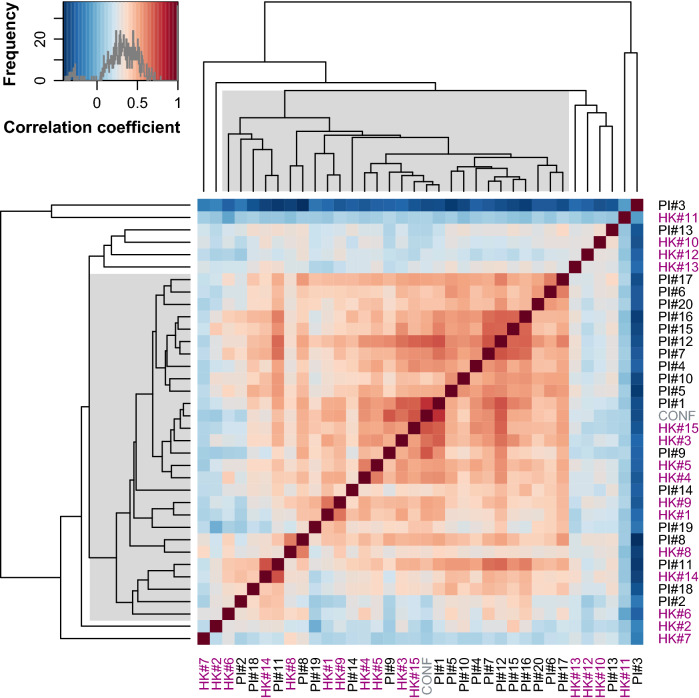
Polychoric correlation matrix and hierarchical clustering (dendrogram) for individual item scores (Survey). Polychoric correlation coefficients are color-coded using the color key shown at the top left histogram. The dendrogram was obtained by a hierarchical clustering based on Pearson correlation distances using the unweighted pair group method with arithmetic mean. CONF, the question pertaining to self-confidence in face recognition ability

The correlation matrix (Fig. 2) generally showed correlations between individual items across the two scales; however, some items were not correlated with other items to the extent that they would reduce the reliability or internal consistency of a single measure pertaining to a single construct. In fact, hierarchical clustering using the unweighted pair group method with arithmetic mean showed that 8 out of 36 items were distant from a cluster to which most items belonged (shaded areas in Fig. 2, dendrogram). These eight items consisted of (Table 2): the four items already known to be irrelevant with respect to face identity recognition (HK#10, HK#11, HK#12, and HK#13), and two items from the HK questionnaire (HK#2 and HK#7), and two items from the PI20 (PI#3 and PI#13). Previous studies reported that five of the eight item-score differences (suspected prosopagnosics − control) were marginal (score difference < 1) between individuals with suspected prosopagnosics and typically developed control individuals (0.45 for HK#10, − 0.39 for HK#11, 0.11 for HK#12, − 0.45 for HK#13, and 0.62 for PI#3) (Kennerknecht et al., 2008; Shah, Gaule, et al., 2015). However, it should be noted that the score difference exceeded 1 for the remaining three items (1.46 for HK#2, 1.12 for HK#7, and 1.16 for PI#13), suggesting that these three items could measure traits that differ from those measured via the other 28 items.

**Table 2:** Test items shown with the mean scores (*N* = 855, survey)

Test item		Mean score (1 SD)	
		Females	Males
HK#1 *	I can easily follow actors in a movie	2.94 (1.21)	2.83 (1.14)
HK#2	Some of my family have problems in recognizing faces	1.49 (0.96)	1.48 (0.89)
HK#3	People often tell me I do not recognize them	2.48 (1.19)	2.49 (1.21)
HK#4 *	I can decide immediately if a face is familiar	2.48 (1.08)	2.41 (1.08)
HK#5	It takes me a long time to recognize people	2.83 (1.14)	2.74 (1.14)
HK#6 *	I always recognize family members	1.15 (0.53)	1.15 (0.58)
HK#7 *	I can easily form a mental picture of a red rose	1.65 (0.86)	1.95 (1.06)
HK#8 *	I can easily form pictures of close friends in my mind	1.56 (0.87)	1.60 (0.91)
HK#9 *	I recognize famous people immediately	2.59 (1.19)	2.59 (1.15)
HK#10 *	I can decide immediately whether a face is male or female	1.93 (0.92)	1.87 (0.78)
HK#11	I get lost in new places	3.77 (1.24)	3.07 (1.28)
HK#12 *	I can see if a face is attractive	2.51 (1.08)	2.42 (1.07)
HK#13	I have problems reading emotions in a face	2.42 (1.02)	2.53 (1.07)
HK#14	I avoid meetings as I might overlook familiar people	1.79 (0.94)	1.86 (0.99)
HK#15	I do not recognize people the day after a brief meeting	3.09 (1.32)	2.93 (1.32)
CONF *	I am confident that I can recognize faces well compared to others	3.33 (1.16)	3.16 (1.22)
PI#1	My face recognition ability is worse than most people	2.82 (1.09)	2.68 (1.10)
PI#2	I have always had a bad memory for faces	2.11 (1.08)	2.06 (1.02)
PI#3	I find it notably easier to recognize people who have distinctive facial features	4.23 (0.78)	4.29 (0.78)
PI#4	I often mistake people I have met before for strangers	2.49 (1.17)	2.51 (1.22)
PI#5	When I was at school I struggled to recognize my classmates	2.05 (1.11)	1.92 (1.02)
PI#6	When people change their hairstyle, or wear hats, I have problems recognizing them	2.69 (1.16)	2.71 (1.16)
PI#7	I sometimes have to warn new people I meet that I am ‘bad with faces’	1.76 (1.13)	1.72 (1.09)
PI#8 *	I find it easy to picture individual faces in my mind	2.45 (1.12)	2.31 (1.01)
PI#9 *	I am better than most people at putting a ‘name to a face’	3.47 (1.15)	3.25 (1.19)
PI#10	Without hearing people's voices, I struggle to recognize them	2.06 (1.01)	2.15 (1.02)
PI#11	Anxiety about face recognition has led me to avoid certain social or professional situations	1.86 (1.08)	1.86 (1.04)
PI#12	I have to try harder than other people to memorize faces	2.33 (1.18)	2.34 (1.19)
PI#13 *	I am very confident in my ability to recognize myself in photographs	1.84 (0.95)	1.97 (1.00)
PI#14	I sometimes find movies hard to follow because of difficulties recognizing characters	2.23 (1.19)	2.08 (1.11)
PI#15	My friends and family think I have bad face recognition or bad face memory	1.79 (1.04)	1.76 (0.98)
PI#16	I feel like I frequently offend people by not recognizing who they are	1.75 (0.97)	1.71 (0.96)
PI#17 *	It is easy for me to recognize individuals in situations that require people to wear similar clothes (e.g. suits, uniforms and swimwear)	2.67 (1.13)	2.53 (1.14)
PI#18	At family gatherings, I sometimes confuse individual family members	1.66 (0.97)	1.63 (0.94)
PI#19 *	I find it easy to recognize celebrities in ‘before-they-were-famous’ photos, even if they have changed considerably	3.44 (1.07)	3.46 (1.06)
PI#20	It is hard to recognize familiar people when I meet them out of context (e.g. meeting a work colleague unexpectedly while shopping)	2.63 (1.23)	2.53 (1.23)

Items marked with asterisks (*) are reverse scored

#### Scale reliability

We found that the reliability coefficients for the PI20 were higher relative to those for the HK11 [HK11: *α* = 0.8449 (95% CI 0.8273, 0.8633), *ω*_*t*_ = 0.8767 (95% CI 0.8571, 0.8880); PI20: *α* = 0.9174 (95% CI 0.9102, 0.9249), *ω*_*t*_ = 0.9368 (95% CI 0.9300, 0.9424)]. Follow-up Feldt paired tests (Feldt, 1980) confirmed significant differences in reliability coefficient between the HK11 and PI20 (difference in *α*: *t*_853_ = 16.4437, *p* = 5.5132 × 10^−53^; difference in *ω*_*t*_: *t*_853_ = 17.4868, *p* = 9.4696 × 10^−59^).

However, the difference in reliability coefficients may merely reflect the difference in the number of items in the questionnaires (Cortina, 1993). To examine this possibility, we compared reliability coefficients of the two questionnaires with a virtual match of the numbers of items (see Data analysis). The brute-force calculation of reliability coefficients showed that the coefficients for the 11-item PI20 subsets were almost comparable (within 1 SD) to those for the HK11 [*α*: mean = 0.8530 ± 0.0392 (± 1 SD), median 0.8474, range 0.7495–0.9324; *ω*_*t*_: mean = 0.8914 ± 0.0225 (± 1 SD), median 0.8933, range 0.8122–0.9438], indicating that the HK11 and PI20 demonstrated almost equivalent reliability at the individual-item level.

### Discussion

These results showed that the two representative face recognition questionnaires are closely related to each other in terms of correlation analyses, PCA, hierarchical clustering, and item reliability. It is worth noting that a recent meta-analysis showed that test–retest reliabilities for instantaneously administered tests are about *r* = 0.8 (Calamia, Markon, & Tranel, 2013), which is comparable to our findings (*r* = 0.8228). This may indicate that the correlation coefficient between the two questionnaires is sufficiently high to consider that the two questionnaires might measure essentially the same trait to the extent of reliability that solid neuropsychological tests can achieve. However, residual variance is not yet trivial in the present case, as 32% of the variance (1 − 0.8228^2^) remains unexplained. It is possible that one questionnaire has a stronger relationship with actual behavioral performance than the other. In experiment, we examined this issue by comparing correlations of HK11 and PI20 with actual face recognition performance.

## Experiment

### Materials and methods

#### Participants

All participants were recruited from job and volunteer web sites for students in Tokyo area. The recruitment advertisement did not ask whether they have difficulty recognizing faces. Neither inclusion nor exclusion criteria are related to self-reported face recognition ability. One hundred and eighty young Japanese adults [81 female, 99 male; mean age: 20.8 ± 1.7 (± 1 SD) years; range 18–27 years] participated in the experiment. All had normal or corrected-to-normal vision and none reported a history of neurological or developmental disorders. All participants received monetary compensation for their 3-h participation along with another psychological experiments [3000 yen or 4000 yen (after an increase in the internal minimal wage)]. No one participated in the survey.

### Procedure

We used TFMT (Cheng et al., 2016), an East Asian face version of CFMT (Duchaine & Nakayama, 2006). The TFMT task was performed using the standard CFMT procedure (Cheng et al., 2016; Shah, Gaule, et al., 2015). In brief, we asked participants to memorize 6 target faces and tested with the same image in Stage 1 (18 trials), to memorize the same six faces and tested with novel images (new viewpoint and/or lighting from the learned faces) in Stage 2 (30 trials), and to memorize the same six faces and tested with novel images (new viewpoint and/or lighting from the learned faces) with visual noise in Stage 3 (24 trials).

Each stage consisted of learning and test phases. In stage 1, the three study images (left 1/3 profile, frontal view, and right 1/3 profile) were presented for 3 s each, then participants were required to perform a three-alternative-forced-choice (3AFC) task, in which three test faces (one target and two distractor faces) were presented and participants were instructed to select the individual whom they were just shown. This procedure was repeated for six target faces (6 target faces × 3 views). In stage 2 and 3, the six target faces (a frontal view) were presented for 20 s, then participants were required to perform 30 (6 target faces × 5 presentations in Stage 2) or 24 (6 target faces × 4 presentations in Stage 3) 3AFC test trials. The experiment took about 15 min to complete. After the experiment, participants completed the questionnaires as in the survey.

### Results

#### Face recognition performance

Mean face recognition performance was 80.11% (± 1 SD = 12.91). Two-sample *t* test showed that female performance [84.36% (± 1 SD = 11.13)] was significantly greater than male performance [76.64% (± 1 SD = 13.28)] (*t*_178_ = 4.1691, *p* = 4.7684 × 10^−5^, Cohen's *d* = 0.6246 (95% CI 0.3230, 0.9245)].

#### Relationship between questionnaire score and face recognition performance

**Fig. 3:**
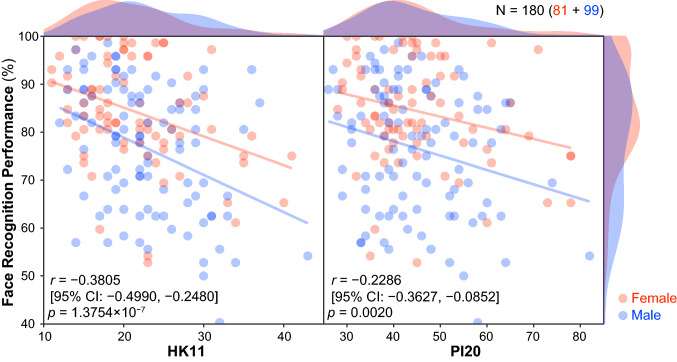
Correlation between self-reported face recognition ability (total scores for the PI20 and HK11) and behavioral face recognition performance (Experiment). Scatter plot with color-coded transparent density curves of total questionnaire scores (*x*-axis; left panel, HK11; right panel, PI20) and behavioral performance on the TFMT (*y*-axis). Dots represent individual data, and color represents sex (red, female; blue, male). The transparent lines represent linear regression lines for each sex using ordinary least squares

Figure 3 shows correlations between questionnaire scores and behavioral face recognition performance. We found significant correlations between HK11 scores and behavior [*r* =  − 0.3805 (95% CI − 0.4990, − 0.2480), *p* = 1.3754 × 10^−7^], and between PI20 scores and behavior [*r* =  − 0.2286 (95% CI − 0.3627, − 0.0852), *p* = 0.0020]. Back-transformed average Fisher's z procedure (Hittner, May, & Silver, 2003) with Zou’s CI showed that the correlation between questionnaire score and behavior was significantly larger for the HK11 than the PI20 [*r*_diff_ = 0.1519 (95% CI 0.0686, 0.2370), *z* = 3.5569, *p* = 0.0004]. There were no significant correlations between the dummy items and behavior [HK#10, *r* =  − 0.0566 (95% CI − 0.2012, 0.0905), *p* = 0.4508; HK#11, *r* = 0.0047 (95% CI − 0.1417, 0.1508), *p* = 0.9502; HK#12, *r* =  − 0.0582 (95% CI − 0.2027, 0.0888), *p* = 0.4378; HK#13, *r* =  − 0.1047 (95% CI − 0.2472, 0.0422), *p* = 0.1617].

Note that Fisher’s *z* test with Zou’s CI showed no significant sex difference in the score–behavior correlation in both the HK11 [*r*_diff_ = 0.0110 (95% CI − 0.2438, 0.2715), *z* = 0.0834, *p* = 0.9335; *r*_females_ =  − 0.3586 (95% CI − 0.5351, − 0.1522), *p* = 0.0010; *r*_males_ =  − 0.3696 (95% CI − 0.5285, − 0.1858), *p* = 0.0002] and the PI20 [*r*_diff_ =  − 0.0076 (95% CI − 0.2828, 0.2723), *z* =  − 0.0529, *p* = 0.9578; *r*_females_ =  − 0.2508 (95% CI − 0.4443, − 0.0338), *p* = 0.0242; *r*_males_ =  − 0.2427 (95% CI − 0.4200, − 0.0476), *p* = 0.0155].

### Discussion

These results suggest that people have modest insight into their face recognition ability and that the HK11 may be a better questionnaire to assess actual face recognition ability than the PI20. Note also that the significant correlation between insight and behavior was specific to the two questionnaire scores that are related to face recognition; we did not find any significant correlations between the dummy items and behavior. This result provides evidence that it is insight into face recognition ability, but not insight into other face processing abilities (i.e., to judge facial gender, attractiveness, or emotion), that has a significant relationship with face identity recognition performance.

While these results indicate a moderate relationship between self-reported face recognition ability and actual behavior, we cannot rule out the possibility that the order (first completed a face recognition task, and then completed the questionnaires) is likely to affect the relationship. Because people could get some insight into their face recognition ability through the experiment, that may eventually lead to an inflation of the correlation. This issue requires further investigation; however, it is notable that the lowest (but significant) correlation coefficient (*r* =  − 0.14) is reported using the same order (Palermo et al., 2017). Other studies using the opposite order (first questionnaire, and then task) even reported somewhat higher correlations, which range from *r* = 0.36 (Bobak, Mileva, & Hancock, 2019) to *r* = 0.44 (Arizpe et al., 2019) (Note that absolute value, not sign, is important). Although the different questionnaires are used in these studies, the order may not be a critical factor that affects the relationship between insight into face recognition ability and behavior.

Although females and males scored similarly in the survey, females showed better behavioral performance than males in the experiment. The behavioral results are expected since a large number of studies have shown superior face processing performance in females (Bobak, Pampoulov, & Bate, 2016; Cellerino, Borghetti, & Sartucci, 2004; Lewin & Herlitz, 2002; Matsuyoshi et al., 2014; McBain, Norton, & Chen, 2009; Shapiro & Penrod, 1986). However, it is surprising that females did not show higher self-reported face recognition ability in the questionnaires, even though they actually excelled at behavioral performance. Although the exact mechanisms remain unclear and it is beyond the scope of our study, the tendency to form same-gender friendship may lead to the comparable self-reported face recognition ability between sexes. Because about 70% of friendships are formed within gender (Reeder, 2003), females may not have enough opportunities to know their superior face recognition ability compared to males (and vice versa) in their daily lives. Furthermore, social factors such as modesty norm (Smith & Huntoon, 2014) might cause females to underrate their ability. Alternatively, the questionnaire items themselves are not simply accurate enough to capture people’s face recognition ability. These explanations are not mutually exclusive, and the cause may differ across individuals; in any case, further investigation is necessary to better understand the females’ underestimation of their ability.

## General discussion

Whether people have insight into their face recognition abilities has been debated recently. Although recent studies reported that people have good insight into their face recognition ability using PI20 (Livingston & Shah, 2017; Shah, Gaule, et al., 2015), other studies showed that people have modest insight using the HK questionnaire (Bobak et al., 2019; Murray, Hills, Bennetts, & Bate, 2018; Palermo et al., 2017). Since the difference might be due to the difference in the questionnaire and/or the bias induced by including an extreme group, we examined the relationship between self-reported face recognition ability and actual behavioral performance using both questionnaires. Our results showed that both questionnaire scores moderately correlated with behavioral face recognition performance (about *r* = 0.3) and that the correlation was stronger for HK11 than for PI20. This suggests that people have modest, not good, insight into their face recognition ability and necessitates a revision of the view that the PI20 overcomes the weakness of the pre-existing questionnaire.

Although the Kennerknecht’s HK questionnaire was criticized because of its “weak relationship” to actual face recognition performance (Shah, Gaule, et al., 2015), our findings showed a significant correlation between HK11 scores and behavioral performance. This might be partially due to the fact that most studies used the score summed over all 15 items when using the HK questionnaire (Johnen et al., 2014; Kennerknecht et al., 2008; Palermo et al., 2017; Stollhoff et al., 2011), even though it includes the four dummy questions. Incorporating irrelevant items to a questionnaire not only reduce the reliability, but also reduce the predictability of a questionnaire in behavioral performance. Using the reduced subset of the pre-existing questionnaire (HK11), which excludes the dummy items, we showed that the Kennerknecht’s HK questionnaire may have a greater potential to capture face recognition ability than the PI20.

Furthermore, the use of extreme group approach might cause the inconsistency between studies. Selecting individuals on the basis of (expected) extreme scores of a sample distribution could result in inflated effect size estimates, which in turn leads to inappropriate expectations or conclusions (Preacher et al., 2005). In fact, although the correlation between PI20 scores and behavioral performance was reported to be high (*r* =  − 0.68) (Shah, Gaule, et al., 2015), it decreased remarkably if the data from people with suspected prosopagnosics was excluded (*r* =  − 0.34) (Livingston & Shah, 2017). Studies that have reported the moderate correlations also did not include suspected prosopagnosics in their sample (Bobak et al., 2019; Palermo et al., 2017). In addition, a recent study has reported that people who have been previously informed of their exceptionally high performance (i.e., ‘super-recognizers’ (Russell, Duchaine, & Nakayama, 2009) actually performed well, whereas naïve participants had only moderate insight into their face recognition ability (Bobak et al., 2019). Thus, if the studied population includes those already known to have poor (Shah, Gaule, et al., 2015, b) or good (Bobak et al., 2019) face recognition ability, it may inflate correlation between insight and behavioral performance. It might be difficult to generalize such findings to naïve individuals across the full range of face recognition abilities. One should be careful in these kinds of participants selection biases that can cause circular analysis (i.e., double dipping) whose results statistics inherently depend on the selection criteria (Kriegeskorte, Simmons, Bellgowan, & Baker, 2009).

Surprisingly, our findings are in line with a recent meta-synthesis that showed that the mean correlation between ability self-evaluations and performance was moderate (*M* = 0.29) (Zell & Krizan, 2014). Although individual effects varied from 0.09 to 0.63, the meta-synthesis indicates that people have limited insight into their ability. If the correlation between self-report and behavioral face recognition performance is not so strong in a naïve population, then what do questionnaire-based measures tell us about face recognition? How do we reconcile self-report with objective performance? Unfortunately, there would be no straightforward way to reliably estimate an individual’s face recognition ability or DP risk. Instead of simply asking participants about insight into their face recognition ability, we might have to improve measurements and/or analytical methods, for example by elaborating the design/texts of a questionnaire, extracting latent cognitive factors from a battery of behavioral tests (e.g., Miyake & Friedman, 2012), and creating a reliable predictive model based on a machine learning technique.

In conclusion, our results suggest that the two representative self-report face recognition questionnaires (Kennerknecht et al., 2008; Shah, Gaule, et al., 2015) measured the similar but slightly different traits, and that people have modest, not good, insight into their face recognition ability. Although the HK11 and/or the PI20 may serve as a moderate (albeit non-definitive) measure for estimating face recognition ability and DP risk (Livingston & Shah, 2017), our findings suggest that, contrary to the Shah et al.’s claims, the reliability and validity of the PI20 may be less than that of the pre-existing questionnaire (precisely, the reduced subset, HK11) (Kennerknecht et al., 2008). Given the current state of DP, where neither objective diagnostic criteria nor biological markers have been established (Barton & Corrow, 2016; Susilo & Duchaine, 2013), we might need to focus on creating a reliable face recognition questionnaire (rather than a ‘DP questionnaire’) that can predict behavioral face recognition performance (Arizpe et al., 2019). Alternatively, more exploratory research not only using HK11 and PI20 together or a combination thereof, but also a range of other face processing measures could aid the extraction of latent prosopagnosia traits/dimensions and the development of valid DP taxonomy. In either case, self-report may not be, at least in its current form, a reliable measure for estimating face recognition ability or DP risk as it gives us limited insight into the prediction of naïve individuals’ face recognition performance.

## Data Availability

The datasets generated during and/or analyzed during the current study are available in the github, https://doi.org/10.5281/zenodo.3555599https://github.com/dicemt/matsuyoshi_selfreport_facerecognition.
